# TAKING CHARGE AND FLEXING PROTOCOLS: SNF NURSING STAFF’S UTI IDENTIFICATION

**DOI:** 10.1093/geroni/igae098.3171

**Published:** 2024-12-31

**Authors:** Kimberly Delgado, Donna Roberson

**Affiliations:** East Carolina University College of Nursing, Greenville, North Carolina, United States; East Carolina University, Greenville, North Carolina, United States

## Abstract

Skilled Nursing Facility (SNF) residents are at increased risk of adverse reactions, including death, due to inappropriate antibiotic use, which accounts for up to 75% of all SNF antibiotic prescriptions; the most common indication being urinary tract infection (UTI). The structure of SNFs create barriers to appropriate diagnosis and treatment of UTIs, with limited registered nurse availability and health care providers (HCP) being off-site. The purpose of this study was to describe SNF nursing staff experiences with residents whom they suspected were presenting with a UTI. A better understanding of what takes place prior to HCP contact can assist SNF leadership with improving nursing-led interventions. This qualitative descriptive study used a semi-stratified purposive sample, snowball sampling, and social media recruitment. Single, semi-structured interviews were completed using open-ended questions. Data analysis resulted in three themes: way out of the norm, taking ownership, and flexing protocols. Participants identified SNF residents’ behaviors that were ‘way out of the norm,’ as a key indicator of a UTI. Approaches to care included nursing staff taking ownership, or responsibility, and flexing protocols to prioritize the care and well-being of their residents. SNF nursing staff spend more time with their residents than the HCP, which results in them being heavily involved in the process of UTI identification. Inclusion of SNF nursing staff in future research is critical to the exploration of practices and improving outcomes. Research study recruitment barriers illustrate the tremendous need for education among SNF facility leadership to garner support and permission to access facilities.

